# Circadian rhythm mechanism in the suprachiasmatic nucleus and its relation to the olfactory system

**DOI:** 10.3389/fncir.2024.1385908

**Published:** 2024-03-25

**Authors:** Yusuke Tsuno, Michihiro Mieda

**Affiliations:** Department of Integrative Neurophysiology, Graduate School of Medical Sciences, Kanazawa University, Kanazawa, Japan

**Keywords:** circadian rhythm, suprachiasmatic nucleus, *in vivo*, olfaction, arginine vasopressin (AVP)

## Abstract

Animals need sleep, and the suprachiasmatic nucleus, the center of the circadian rhythm, plays an important role in determining the timing of sleep. The main input to the suprachiasmatic nucleus is the retinohypothalamic tract, with additional inputs from the intergeniculate leaflet pathway, the serotonergic afferent from the raphe, and other hypothalamic regions. Within the suprachiasmatic nucleus, two of the major subtypes are vasoactive intestinal polypeptide (VIP)-positive neurons and arginine-vasopressin (AVP)-positive neurons. VIP neurons are important for light entrainment and synchronization of suprachiasmatic nucleus neurons, whereas AVP neurons are important for circadian period determination. Output targets of the suprachiasmatic nucleus include the hypothalamus (subparaventricular zone, paraventricular hypothalamic nucleus, preoptic area, and medial hypothalamus), the thalamus (paraventricular thalamic nuclei), and lateral septum. The suprachiasmatic nucleus also sends information through several brain regions to the pineal gland. The olfactory bulb is thought to be able to generate a circadian rhythm without the suprachiasmatic nucleus. Some reports indicate that circadian rhythms of the olfactory bulb and olfactory cortex exist in the absence of the suprachiasmatic nucleus, but another report claims the influence of the suprachiasmatic nucleus. The regulation of circadian rhythms by sensory inputs other than light stimuli, including olfaction, has not been well studied and further progress is expected.

## Introduction

Sleep is essential for survival, and animals maintain their physical and mental health by maintaining a certain rhythm of waking and sleeping at the right time and in the right state. In humans, waking and sleeping roughly correspond to the day and night in the outside world. Diurnal organisms, such as humans, are active during the day, while nocturnal animals, such as mice, are more active at night and sleep more at the opposite time. The suprachiasmatic nucleus (SCN) is the center that controls all circadian rhythms, including wake–sleep, appetite, autonomic system, and neuroendocrine rhythms (Hastings et al., 2018). This control of circadian rhythms allows animals to maintain a roughly 24-h cycle, even in the absence of light from the outside world. The circadian rhythm period of the body clock is approximately 24 h, not exactly 24 h, so each morning’s exposure to light resets the body clock and aligns it with the external day-night cycle. In this review, we will discuss the inputs to the SCN, the networks within the SCN, the outputs from the SCN, and the effects of stimuli other than light on circadian rhythms, particularly the olfactory system.

## Inputs to the SCN

**Figure 1:**
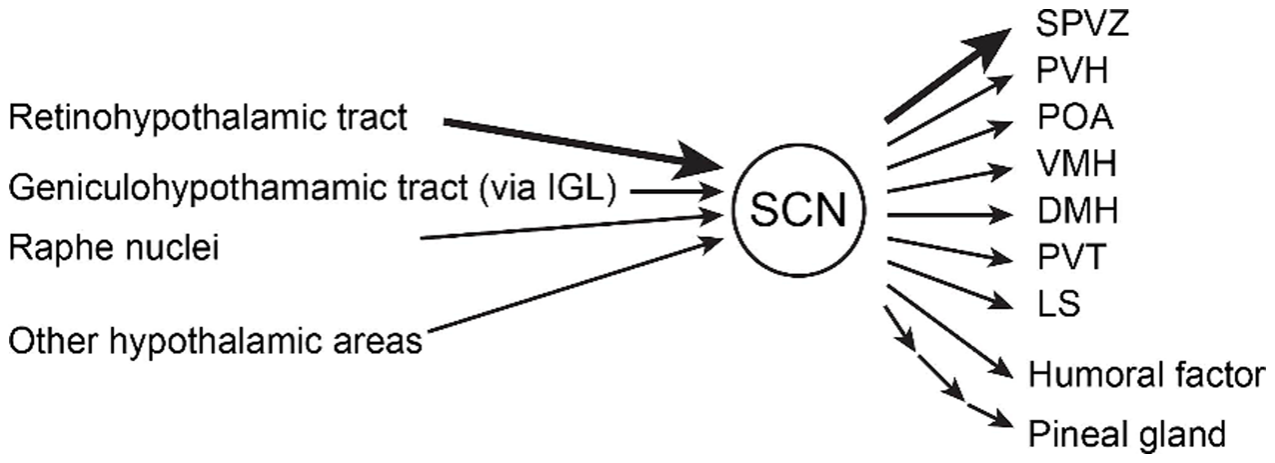
Inputs and outputs of the suprachiasmatic nucleus (SCN). The major inputs to the SCN are (1) the retinohypothalamic tract from melanopsin-containing photosensitive ganglion cells in the retina, (2) the geniculohypothalamic tract via the intergeniculate leaflet (IGL), and (3) the median raphe nuclei containing serotonergic neurons. Other hypothalamic areas (arcuate nucleus, medial preoptic area) and thalamic areas (paraventricular thalamic nuclei) also project to the SCN. Humoral factors such as hormones also modulate the SCN. The major outputs of the SCN are (1) subparaventricular zone (SPVZ), (2) paraventricular hypothalamic nucleus (PVH), (3) preoptic area (POA), (4) ventromedial hypothalamic nucleus (VMH), (5) dorsomedial hypothalamic nucleus (DMH), (6) paraventricular thalamic nuclei (PVT), and (7) lateral septum (LS). Other outputs include humoral factor and pineal gland polysynaptically (Abrahamson and Moore, 2001; Saper et al., 2005; Starnes and Jones, 2023).

There are three major inputs to the SCN (Abrahamson and Moore, 2001; Reghunandanan and Reghunandanan, 2006; Morin, 2013) (Figure 1). The most important input is from the retina through the retinohypothalamic tract (RHT) to the ventral core region of the SCN which is thought to provide input primarily to vasoactive intestinal polypeptide (VIP)-positive and gastrin-releasing peptide (GRP)-positive neurons (Lokshin et al., 2015). Loss of melanopsin-containing intrinsically photosensitive retinal ganglion cells (ipRGCs, Opn4+) that form the RHT, abolishes circadian photoentrainment (Güler et al., 2008), suggesting that ipRGCs are essential for photoentrainment. The major neurotransmitters are thought to be glutamate and PACAP (Hannibal, 2002, 2021), and the glutamatergic input is mediated by AMPA and NMDA receptors (Kim and Dudek, 1991).

The second is the geniculohypothalamic input via the thalamic intergeniculate leaflet, which also originates from the retina. The third is serotonergic input from the raphe nuclei, which enters the ventral region of the SCN (Abrahamson and Moore, 2001). Serotonin receptors (5-HT_1A, 2A, 2B, 2C, 5A, 7_) are expressed in the SCN (Moyer and Kennaway, 1999; Takeuchi et al., 2014). Other inputs come from other hypothalamic areas that can modulate the activity of the SCN. The circadian rhythm of the SCN is thought to be altered by sleep, wakefulness, animal state, and external conditions but either the pathway and mechanism are unknown (Deboer et al., 2003, 2007).

## Inside the SCN

The two of the major neuronal subtypes in the SCN are VIP-positive neurons in the ventrolateral region, also known as the core region, and AVP-positive neurons in the dorsomedial, or shell, region (Abrahamson and Moore, 2001). VIP-positive neurons receive direct projection from the retina (Todd et al., 2020) and are thought to shift the phases of the circadian rhythm. Spontaneous Ca^2+^ activity of VIP neurons is higher during daytime and subjective daytime (Jones et al., 2018). VIP neuron intracellular Ca^2+^ is increased by light stimulation of the retina (Jones et al., 2018; Todd et al., 2020). Suppression of VIP neurons abolished the phase shift induced by light pulses (Jones et al., 2018). Diphtheria toxin ablation of VIP neurons disrupted the light-entrained rhythm of locomotor activity (Todd et al., 2020). All of these results suggest that SCN VIP neurons play an important role in light entrainment. In addition, because VIP or VIP receptor, VPAC2, knock-out mice show either arrhythmicity, multiple circadian periods, or a shorter single period in locomotor activity (home-cage activity) or wheel-running activity (Harmar et al., 2002; Colwell et al., 2003; Hughes et al., 2004; Aton et al., 2005; Peng et al., 2022), the VIP peptide itself is important for synchronizing circadian rhythmicity within the SCN and animal behavior.

AVP-positive neurons are important in determining the period of the circadian rhythm. Knockout of the clock gene Bmal1 specifically in AVP neurons impaired the behavioral circadian rhythm (Mieda et al., 2015). The clock proteins CLOCK (circadian locomotor output cycles kaput) and BMAL1 (basic helix–loop–helix ARNT-like protein 1 or ARNTL, aryl hydrocarbon receptor nuclear translocator-like protein 1) are key transcription factors that form a heteromer and bind regulatory elements E-boxes to express the clock proteins PER1, PER2, CRY1, CRY2, which in turn inhibit CLOCK-BMAL1, generating the approximately 24-h rhythm (Takahashi, 2017). This machinery is called the transcriptional translational feedback loop (TTFL), which is the core machinery of the molecular clock. Therefore, knocking out Bmal1 in AVP neurons disrupts the cellular clock of AVP neurons.

Casein kinase 1 delta (CK1δ) regulates the nuclear translocation and degradation of the clock proteins PER and CRY. Therefore, the loss of CK1δ elongates the cellular circadian period (Etchegaray et al., 2009, 2010). AVP neuron-specific knockout of CK1δ prolonged the period of the behavioral circadian rhythm (Mieda et al., 2016). In addition, knockout of CK1δ specifically in AVP neurons prolonged the period of the Ca^2+^ activity circadian rhythm in both AVP neurons and VIP neurons (Tsuno et al., 2023). This suggests that AVP neurons regulate the period of the circadian rhythm of the entire SCN. However, since the period of the circadian rhythm was not altered by loss of GABA release (Maejima et al., 2021) or AVP release (Tsuno et al., 2023) specifically in AVP neurons, it is likely that neurotransmitters other than GABA and AVP are responsible for the period regulation of the SCN ensemble circadian rhythm and locomotion by AVP neurons, and further studies are needed to identify the mechanism.

In addition to AVP neurons and VIP neurons, the SCN contains neurons that express a variety of neuropeptides. For example, CCK-positive neurons in mice have a peak in Ca^2+^ activity 5 h before the onset of locomotor activity under different light/dark conditions, suggesting that they may determine the onset of locomotion (Xie et al., 2023). Prok2-positive neurons have a peak in Ca^2+^ activity in the middle of the day (Onodera et al., 2023), which may inhibit locomotor output (Cheng et al., 2002). Abrahamson and Moore showed that ventral core neurons contain GABA, VIP, gastrin-releasing peptide (GRP), neurotensin (NT), calretinin (CALR), and calbindin (CALB), whereas the dorsal shell neurons contain GABA, AVP, met-enkephalin (mENK), angiotensin II (AII), and CALB (Abrahamson and Moore, 2001). Recent single-cell transcriptomic analyses have shown that the SCN neurons can be classified into 5 (Park et al., 2016; Wen et al., 2020), 11 (Morris et al., 2021) or 12 subtypes (Xu et al., 2021). Park et al. (2016) classified 5 groups of neurons, VIP+, AVP+/VIPR2+, PACAP+/Prok2+, PACAPR+, and others. Wen et al. (2020) classified 5 groups, Vip+/Nms+, Avp+/Nms+, Cck+/C1ql3+, Cck+/Bdnf+, and Grp+/Vip+. Morris et al. (2021) classified 11 neuronal subpopulations, 8 groups in day and 8 groups in night including 5 overlapping groups. Xu et al. (2021) classified 12 groups, 4 AVP+, 3 VIP+, 2 CCK+, and 3 unknown groups. One of 4 AVP+ groups by Xu et al. strongly correlated with the Cck+/C1ql3+ group by Wen et al., while another 3 of 4 AVP+ groups significantly correlated with the Avp+/Nms + group. In addition, 1 of 3 VIP+ groups by Xu et al. correlated with the Vip+/Nms + group by Wen et al., while another 1 VIP+ group correlated with the Grp+/Vip + group. Finally, 1 of 2 CCK+ groups correlated with the Cck+/Bdnf+ group (Wen et al., 2020; Xu et al., 2021). Further studies are needed to elucidate the function of each cell type and the interactions between them.

## Output from the SCN

Output from the SCN is primarily to other hypothalamic nuclei and the thalamus (Abrahamson and Moore, 2001; Saper et al., 2005; Reghunandanan and Reghunandanan, 2006; Morin, 2013; Starnes and Jones, 2023). Many projections from the SCN terminate in the subparaventricular zone (SPVZ, projecting DMH), adjacent to the dorsal SCN. Other major projections include the paraventricular hypothalamic nucleus (PVH, including the pituitary–adrenal axis), the preoptic area [POA, a critical area of thermoregulation (Nakamura et al., 2022) and one of non-REM sleep centers (Scammell et al., 2017)], and other hypothalamic nuclei that control instinctive behavior and homeostasis such as feeding, energy metabolism, sleep/wakefulness, and motivated behavior, including the ventromedial hypothalamic nucleus (VMH, projecting SPVZ), dorsomedial hypothalamic nucleus (DMH), paraventricular thalamic nuclei (PVT), and lateral septum (LS). The SCN inhibits the pineal gland, which produces melatonin with sleep-inducing properties, via the PVH, the intermediolateral nucleus of the medulla (IML), and the cervical sympathetic ganglion (Tonon et al., 2021).

## Olfaction and circadian rhythms

There are reports suggesting a relationship between the olfactory system and circadian rhythms. Almost every cell in the body can generate circadian rhythms, with the SCN acting as a master pacemaker to synchronize and entrain the peripheral clock (Reppert and Weaver, 2002; Takahashi, 2017). The olfactory bulb (OB), the first center of olfactory information processing, is thought to be capable of generating circadian rhythms. In slices, the OB maintains a circadian rhythm of Per1 expression, recorded by the Per1-Luc reporter, and neuronal firing activity in rats (Granados-Fuentes et al., 2004) or Per2 expression by the Per2::Luc reporter in mice (Ono et al., 2015), suggesting that the OB can generate its own circadian rhythm. However, the phase of the rhythm is easily altered by external perturbations, as the peak of Per2::Luc varies with the cutting time (Ono et al., 2015). Furthermore, the SCN lesions alter (Abraham et al., 2005) or eliminate (Ono et al., 2015) the peak phase of clock gene expression in the OB. These results suggest that the OB expresses clock genes and can generate circadian rhythms. However, without the SCN, the peaks become desynchronized with the whole body, indicating a hierarchical structure with the SCN as the central clock.

Some reports suggest that the daily rhythm of the olfactory function is influenced by both the SCN and the OB. Granados-Fuentes et al. reported in 2006 that the cedar oil-induced daily rhythm of c-Fos expression remained in the OB and piriform cortex after SCN removal, whereas it disappeared in the piriform cortex after OB ablation, suggesting that the OB generates the rhythm. Granados-Fuentes et al. (2011) reported that the SCN lesion altered the peak phases of the daily rhythm of olfactory discrimination performance (Granados-Fuentes et al., 2011). In addition, Takeuchi et al. reported in 2023 that knockout of Bmal1 in Vgat-positive neurons, including SCN neurons, altered the peak phase of the circadian rhythm of odor-induced c-fos positive cell number in the piriform cortex (Takeuchi et al., 2023). These results indicate that the SCN alters the peak phase of olfactory function, suggesting that it contributes to the synchronization of the circadian rhythms of olfactory function and the central clock.

Clock gene expression itself is important for odor detection. Granados-Fuentes et al. (2011) reported that there is a daily rhythm in the performance and sensitivity of the odor detection task that disappears when the clock genes *Bmal1* or both *Per1* and *Per2* genes are knocked out (Granados-Fuentes et al., 2011). Takeuchi et al. report that Bmal1 knockout in the piriform cortex abolishes the circadian rhythm of the odor-evoked activity (Takeuchi et al., 2023). Using quantitative PCR, they report that the circadian rhythm of gene expression observed in the piriform cortex is dependent on Bmal1 expression in Emx-positive cells, including pyramidal cells in the piriform cortex (Takeuchi et al., 2023). These results suggest that the circadian rhythm of odor detection and odor-evoked neural activity requires clock genes.

The mechanism by which the SCN modulates the circadian rhythm of the OB is not yet clear. It is known that the SCN projects to various hypothalamic regions and releases neurotransmitters, including neuropeptides, and hormones, while the OB receives centrifugal inputs from the olfactory cortex and neuromodulatory centers and is also influenced by neuropeptides and hormones (Brunert and Rothermel, 2021). Therefore, it is possible that the SCN can alter the circadian rhythm of the OB through centrifugal inputs from the olfactory cortex or neuromodulatory centers via the hypothalamus, neuromodulatory inputs, and neuropeptides, or hormones in the bloodstream.

Conversely, there is some evidence that the olfactory system can modify the activity of the SCN via the OB. Amir et al. (1999) reported that light-induced c-Fos expression in the SCN is enhanced by olfactory stimuli. Removal of the OB also altered the time of re-entrainment and altered the phase of the odor-evoked c-Fos expression rhythm in the SCN (Granados-Fuentes et al., 2006). Because odor stimulation increased c-Fos expression in the paraventricular thalamic nucleus (PVT) (Amir et al., 1999), a review has argued that the PVT may be related to the pathway from the OB to the SCN (Jeffs et al., 2023).

## Discussion

Although the basic mechanisms of circadian rhythm control are becoming clearer, much remains unknown about how the individual subpopulations within the SCN interact to generate a synchronized circadian rhythm as an ensemble. Furthermore, although circadian rhythms are closely linked to the sleep–wake cycle (Franken and Dijk, 2024), the interaction between the SCN and the sleep–wake center remains elusive. Although the modulation of circadian rhythms by light stimulation has been well studied, the modulation of circadian rhythms by environmental changes other than light has not been fully investigated, and further research is needed.

The SCN, the center of the circadian rhythm, modulates the rhythm in response to external conditions. In addition to light, possible conditions include food availability, the presence of predators, and environmental temperature, which in turn affect the wake–sleep states and the brain’s information processing mode (Pickel and Sung, 2020; Franken and Dijk, 2024). From this perspective, the olfactory system, which is important for feeding, finding mates, and escaping predators, is thought to be closely related to circadian rhythms and wake–sleep states (Mori and Sakano, 2022). Because the OB has the unique property of generating a circadian rhythm without the SCN, investigating the relationship between diurnal variation in olfactory information processing and circadian rhythms of the SCN is an interesting target for future research. In addition, a reduced sense of smell and instability of the wake–sleep rhythm are two prominent features of Alzheimer’s disease (AD), so both may be easily impaired and may be an indicator of preclinical AD (Jeffs et al., 2023).

## Author contributions

YT: Conceptualization, Funding acquisition, Writing – original draft, Writing – review & editing. MM: Conceptualization, Funding acquisition, Writing – original draft, Writing – review & editing.
